# MRI-Based Geometric Modeling for Personalized Transcranial Magnetic Stimulation in Age-Related Neurodegenerative Diseases

**DOI:** 10.3389/fnins.2021.685424

**Published:** 2021-07-22

**Authors:** Hanna Lu

**Affiliations:** ^1^Department of Psychiatry, The Chinese University of Hong Kong, Hong Kong, China; ^2^Centre for Neuromodulation and Rehabilitation, The Affiliated Brain Hospital of Guangzhou Medical University, Guangzhou, China

**Keywords:** TMS, personalized medicine, geometry, MRI, modeling, simulation, cortical features

## Introduction

Structural magnetic resonance imaging (MRI) provides a powerful and financially favorable imaging modality, with the additional benefits of its high spatial resolution and plausible multiscale measurements of brain morphometry (Madan and Kensinger, 2016; Lu, 2020). With the use of MRI-based morphometry, region-specific cortical features have been found to enhance the accuracy of diagnosis, and even predict the treatment responses (Bartlett et al., 2018). Of all the advanced modalities of non-invasive brain stimulation (NIBS), transcranial magnetic stimulation (TMS) is an FDA-cleared technique for the treatments of the main types of brain disorders through non-invasive modulation of brain activities using a magnetically induced electric field (E-field) (Rossi et al., 2009). Although using individual MRI images to guide TMS has improved the accuracy of localizing the stimulation targets, the treatment response still greatly varies between individuals, particularly in the patients with age-related neurodegenerative diseases (Polanía et al., 2018). Among the factors that determine the variability of TMS-induced effects, stimulation intensity and individual cortical morphometry were highlighted as brain stimulation-specific factors (Polanía et al., 2018).

## Why Should Computational Modeling Be Considered?

**Figure 1:**
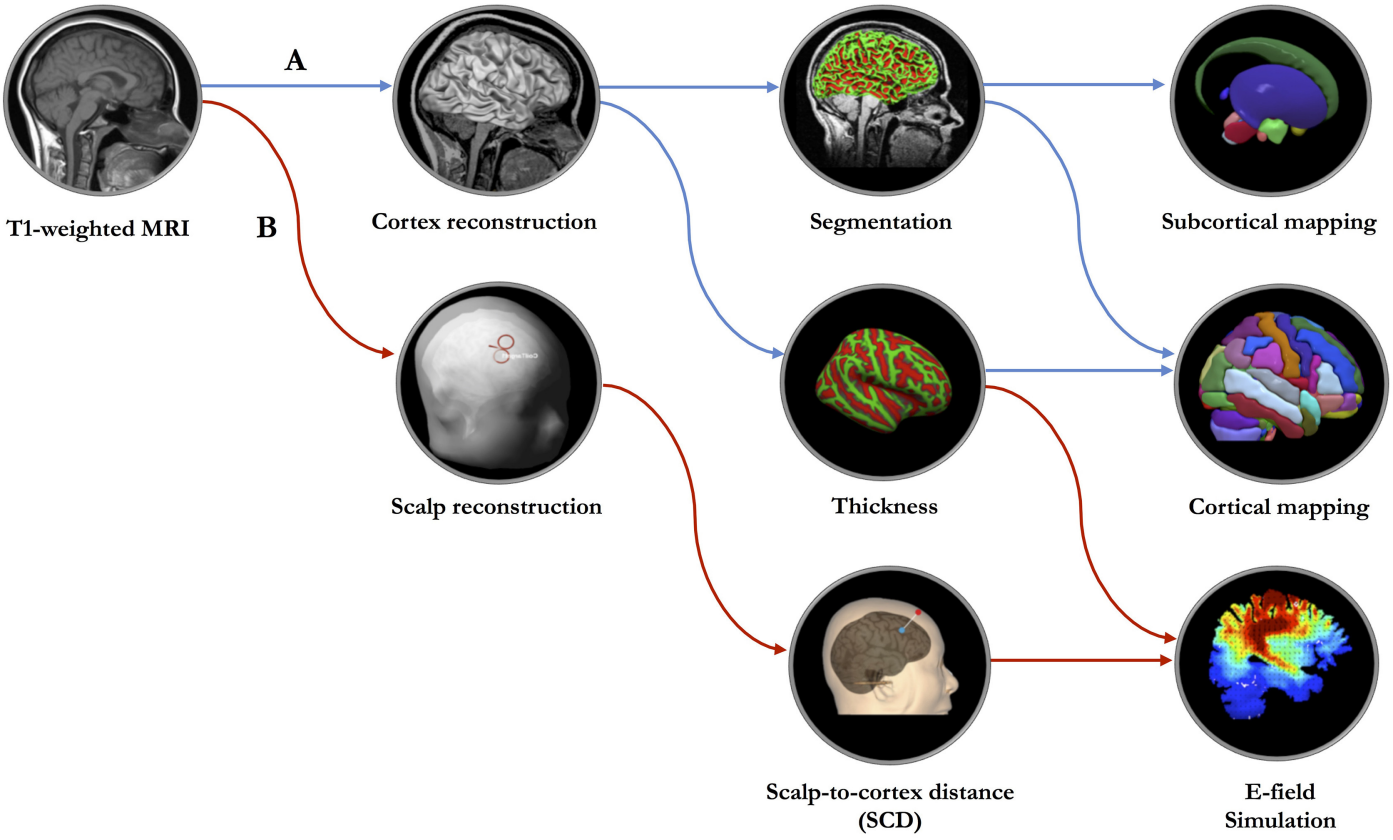
The proposed geometric model of network-based transcranial brain stimulation. **(A)** Based on high-resolution T1-weighted structural MRI data, surface-based morphometry analysis will be performed to measure the cortical and subcortical structures. **(B)** MRI data will be imported to the neuronavigation system for constructing scalp and cerebral cortex, localizing the targeted cortical regions, measuring the scalp-to-cortex distance (SCD) and establishing the simulation model of SCD-dependent electric field (E-field).

In clinical practice, optimized and personalized TMS treatments are impeded by the heterogeneity of cortical morphometry (Caulfield et al., 2020). Fundamental questions persist regarding the landscape of the reconstructed scalp and cortex (Figure 1A) that are based on geometric models of the cortical surface and the identification of the borders between different tissue types. Certainly, the process of reconstruction and how the reconstructed features of scalp and cortex will substantially affect the magnitude and intensity of the TMS-induced E-field, especially when considering the geometry of cortical surface (Polanía et al., 2018). Although volumetric measures have great promise with respect to clinical translation, its low sensitivity to geometric space may limit its utility for navigating brain stimulation. Of note, this challenge has been addressed by recent advances in the analytical methods applicable to quantitatively measure the geometric features of targeted cortex (Lu et al., 2019; Aberra et al., 2020).

Indeed, computational modeling is a powerful tool for examining the biophysical mechanisms of TMS as well as for optimizing the parameters for engaged target and personalized dosage. Previous efforts focused on calculating the spatial distribution of E-field are based on the MRI images of young adults with an average brain size (Lee et al., 2016; Huang et al., 2017). However, this standard model is lack of the representation of the TMS induced E-field in individuals with brain atrophy. Thus, the head model of TMS should be constructed in the combination of the morphometric features capturing the inter- and intra-individual variability related to the stimulation targets.

## The Importance of Geometric Features

To optimize the TMS protocols, a geometric measure, also a key parameter of NIBS, scalp-to-cortex distance (SCD), combined with cortical thickness, should be considered in the construction of head model for the individuals with age-related neurodegenerative diseases, such as Alzheimer's disease (AD) (Figure 1B). The importance of combining SCD and cortical thickness in the geometric model can be explained from three aspects: (1) Location: SCD is a vector-like indicator that links the point on the scalp to the point on the cortical surface, gyrus in particular. Cortical thickness is calculated as an average of the distance from the inner surface of gyrus to the closest point on the outer surface of gyrus and from that point back to the closest point to the inner surface of gyrus, consisting of six layers featured with cytoarchitectonic subdivisions (Amunts et al., 2013). Regarding the mechanisms of TMS (Terao and Ugawa, 2002), interneurons embedded in different cortical layers are the targets related to specific neuropsychiatric symptoms or cognitive dysfunction. Thus, the combination of scale-dependent geometric features (i.e., mm) might be critical to accurately modulate the activities of layer-specific neurons. (2) Dimension: After reconstructing the scalp and cortex, the locations of predefined stimulation targets are commonly determined by the coordinates in three-dimensional (3D) space, such as Montreal Neurological Institute (MNI) space. It should be noted that except for the three spatial dimensions (i.e., *x, y, z*) of stimulation target, the orientation of the TMS coil has another three rotational dimensions, that are roll (*x*_coil_), pitch (*y*_coil_), and yaw (*z*_coil_). Presumably, thus, the precise measurement of the SCD also reflects the optimal placement of the TMS coil with six dimensions correspondingly. (3) Dosage: When evaluating the treatment response of TMS, dosing is one of the fundamental and key variables that varies in individuals (Lisanby, 2017). Generally, the TMS system is capable of reliably and quickly determining the amount of electromagnetism each individual needs to detect the electromyography (EMG) threshold levels in the thumb through single-pulse TMS to stimulate the primary motor cortex (M1) (i.e., motor threshold). Later, this value will be used to determine the TMS output of the targeted regions, such as dorsolateral prefrontal cortex (DLPFC). Prior evidence has shown that the motor threshold is highly dependent on the SCD of M1 (Stokes et al., 2007). Moreover, the observed differences of the SCDs between M1 and DLPFC across disease-specific populations raise the concerns about the existence of the individual variability of TMS dosage (Lu et al., 2019).

To sum up, a high-resolution structural MRI is an invaluable and cost-effective imaging modality that has enormous potential in studies of brain stimulation. Quantitative measurements of stimulation target-related morphometric and geometric features demonstrate a promising utility for improving the current head model of TMS.

## Future Directions

Based on high-resolution MRI data, TMS treatment will eventually be conducted and evaluated at the individual level. Importantly, a novel approach named reverse-calculation E-field allows researchers to transfer traditional fixed dosage based on published studies to personalized dosage based on a computational model (Caulfield et al., 2020). Beyond imaging modality, a multiscale head model that is incorporated with geometric features can improve the localization of stimulated region, increase the power of TMS effects, and open the gate of in-depth understanding the mechanisms of TMS in neurodegenerative diseases.

## Author Contributions

The author confirms being the sole contributor of this work and has approved it for publication.

## Conflict of Interest

The author declares that the research was conducted in the absence of any commercial or financial relationships that could be construed as a potential conflict of interest.
